# Multispectral imaging using a single bucket detector

**DOI:** 10.1038/srep24752

**Published:** 2016-04-22

**Authors:** Liheng Bian, Jinli Suo, Guohai Situ, Ziwei Li, Jingtao Fan, Feng Chen, Qionghai Dai

**Affiliations:** 1 Department of Automation, Tsinghua University, Beijing 100084, China; 2 Shanghai Institute of Optics and Fine Mechanics, Chinese Academy of Sciences, Shanghai 201800, China

## Abstract

Existing multispectral imagers mostly use available array sensors to separately measure 2D data slices in a 3D spatial-spectral data cube. Thus they suffer from low photon efficiency, limited spectrum range and high cost. To address these issues, we propose to conduct multispectral imaging using a single bucket detector, to take full advantage of its high sensitivity, wide spectrum range, low cost, small size and light weight. Technically, utilizing the detector’s fast response, a scene’s 3D spatial-spectral information is multiplexed into a dense 1D measurement sequence and then demultiplexed computationally under the single pixel imaging scheme. A proof-of-concept setup is built to capture multispectral data of 64 pixels × 64 pixels × 10 wavelength bands ranging from 450 nm to 650 nm, with the acquisition time being 1 minute. The imaging scheme holds great potentials for various low light and airborne applications, and can be easily manufactured as production-volume portable multispectral imagers.

Multispectral imaging is a technique capturing spatial-spectral data cubes of scenes, which contain a set of 2D images under different wavelengths. With both spatial and spectral resolving abilities, it is extremely useful and vital for surveying scenes and extracting detailed information^1^. Existing multispectral imagers mostly utilize dispersive optical devices (e.g., prism and diffraction grating) or narrow band filters to separate lights of different wavelengths, and then use an array detector to record them separately^2^,^3^,^4^. Utilizing compressive sensing techniques, multispectral images can be multiplexed together to reduce requisite number of shots^5^. Another kind of multispectral imaging method is the Fourier spectroscopy technique^6^. This approach uses an interferometer to divide the incoming beam into two halves, and change their optical path difference to generate varying interference intensities at each spatial point. Then spectral information can be extracted by applying Fourier transform to these intensities measured by an array detector. Despite diverse principles and setups of the above multispectral imaging methods, photons are detected separately either in the spatial or spectral domain using array detectors. Therefore, these multispectral imagers are photon inefficient and spectrum range limited. Besides, they are usually bulky^4^ and highly expensive (for example, more than $50,000 for a NIR-SWIR multispectral imager^7^). These disadvantages prevent them from wide practical applications.

Differently, single pixel imaging (SPI)^8^,^9^ provides a promising scheme being able to address the above issues of existing multispectral imaging instruments. Using a bucket detector instead of expensive and bulky CCD or CMOS, SPI systems are low cost, compact, and own wider spectral detection range^10^. Besides, SPI collects all the lights interacted with the target scene into a single detection unit, and thus is more photon efficient^11^,^12^,^13^. What’s more, SPI is flexible, meaning that it attaches no requirement on the light path between the target scene and the detector, providing that all the interacted lights arrive at the detector^14^. In the past years, SPI has achieved great success in 2D imaging and various applications^15^,^16^,^17^,^18^,^19^,^20^.

To produce the advantages of SPI in multispectral imaging, there are two intuitional ways. One is to resolve the spectra of collected lights at the detector. Existing such methods include i) directly replacing the bucket detector with a spectrometer^21^,^22^, and ii) using light filters^23^,^24^ or dispersive optical devices^10^,^25^ to separate lights of different wavelengths before separately measuring them. Another straightforward way is to directly extend the 2D spatial modulation in conventional SPI to 3D spatial-spectral modulation using two spatial light modulators. However, this would largely increase requisite projections^11^ and corresponding computation complexity for reconstruction. In a word, since a single bucket detector cannot distinguish different spectra, the above methods need either high commercial cost or geometrically increased projections and computational cost for multispectral imaging.

In this paper, we propose a novel multispectral imaging technique utilizing SPI, termed as multispectral single pixel imaging (MSPI), without increasing requisite projections and capturing time compared to conventional SPI. The main difference between MSPI and conventional SPI is illustrated in Fig. 1. Utilizing the fact that the response speed of a bucket detector (MHz or GHz) is magnitudes faster than that of a spatial light modulator (no higher than KHz)^15^,^23^,^26^, we encode the target scene’s spectral information into this speed gap. Specifically, the proposed MSPI technique introduces spectrum-dependent sinusoidal intensity modulation to illumination during the elapse of each spatially modulated pattern. Thus, different spectrum bands are multiplexed together into a 1D dense measurement sequence of the bucket detector in a frequency-division multiplexing manner. Since the response signals of different wavelength bands display distinct dominant frequencies in the Fourier domain, we conduct a simple Fourier decomposition to separate the multispectral response signals from each other. Then a compressive sensing algorithm^8^ is separately applied to these signals of different wavelength bands to reconstruct the latent multispectral images. The spectral multiplexing and demultiplexing based on Fourier decomposition can suppress system noise effectively, and thus produces high robustness to measurement noise and ensures high reconstruction quality.

MSPI owns a lot of potential applications in various fields of science. Due to its high photon efficiency and robustness to noise which benefit from the spatial-spectral multiplexing and demultiplexing^27^, MSPI owns more advantages when used in low light conditions, such as fluorescence microscopy^28^ and Raman imaging^12^. Besides, the utilized SPI scheme enables MSPI systems to be of compact size and low weight compared to conventional multispectral imagers. This is beneficial for a lot of airborne applications, including geologic mapping, mineral exploration, agricultural assessment, environmental monitoring, and so on^29^. Moreover, MSPI applies to a wide spectral range and is of low cost (the same price level as commercial projectors), and thus can be manufactured as production-volume portable devices for daily use.

## Results

### Experimental setup

MSPI builds on the SPI scheme. In SPI, the incident uniform illumination from a bulb is patterned by a spatial light modulator (SLM), and then projected onto the target scene to encode its spatial information. Simultaneously, a bucket detector is used to record the multiplexed lights. Finally, a compressive sensing algorithm^8^ is applied to retrieve the scene’s spatial information. Under a similar architecture, MSPI adds additional spectral modulation to the incident illumination patterns to further resolve the target scene’s spectral information. Integrating both the spatial and spectral modulation, MSPI can resolve a spatial-spectral 3D data cube of the target scene from the bucket detector’s 1D measurement sequence.

**Figure 2:**
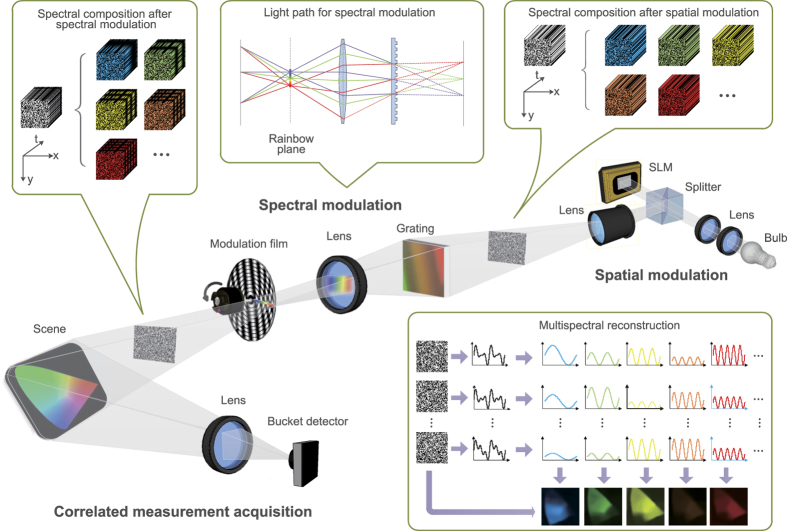
Schematic of the proposed multispectral single pixel imaging (MSPI) system. The broadband light from a bulb is spatially modulated by a spatial light modulator (SLM) to generate a series of 2D random illumination patterns. Next, the spectra of these 2D patterns are stretched into a rainbow stripe using a diffraction grating and a set of lenses. Then the rainbow spectra are modulated by a rotating film before transformed back to 2D spatial patterns. After both the spatial and spectral modulation, the incident illumination is tailored structurally in three dimensions—random in the 2D spatial dimensions and sinusoidal along the spectral dimension. Then the patterns illuminate the target scene to encode both its spatial and spectral information. Finally a bucket detector is utilized to record the correlated lights. In the subsequent reconstruction process, different spectral response signals are decoded by Fourier decomposition, while the spatial information are demodulated by a compressive sensing based reconstruction algorithm. Details of the modulations and demodulations are shown in the insets.

We built a proof-of-concept setup to validate the functionality of MSPI, as shown in Fig. 2. A broadband light source (Epson white 230 W UHE lamp) is converged and collimated via a set of optical lenses for succeeding modulation. For spatial modulation, we use a digital micromirror device (DMD, Texas Instrument DLP Discovery 4100 DLP7000), which can spatially modulate incident light and switch binary patterns at a given frequency (20 kHz of maximum) with clean-cut pattern transition. At this stage, the spatial illumination pattern’s light intensity is temporally constant, as visualized in the top right inset in Fig. 2. Then the illumination pattern goes through a projector lens (Epson, NA 0.27) for subsequent spectral modulation. The spectral modulation module is similar to the agile multispectral optical setup in ref. 30, with the light path displayed in the top middle inset. Specifically, an optical transmission grating (600 grooves, 50 *mm* × 50 *mm*) is placed on the focal plane of the illumination pattern. Then a convex lens collects the first order dispersed spectrum from the grating, and focuses it onto the rainbow plane, where a round film printed with sinusoidal gray annuluses owning different periods is placed for spectral modulation. The rainbow spectrum stretches along the film’s radius. Driven by an electric motor rotating at a constant speed (around 6000 r/min), the film realizes a wavelength-dependent sinusoidal intensity modulation to the spectrum, i.e., different wavelength bands own different temporally sinusoidal intensity variations, as visualized in the top left inset of Fig. 2. After both the spatial and spectral modulation, the illumination pattern interacts with the target scene, and the correlated lights are recorded by a bucket detector (Thorlabs PDA100A-EC Silicon photodiode, 340–1100 *nm*) together with a 14-bit acquisition board (ART PCI8514). For reconstruction, as displayed in the bottom right inset of Fig. 2, we first conduct spectral demultiplexing using Fourier decomposition (fast Fourier transform whose computation complexity is 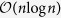), and then reconstruct multispectral images using the linearized alternating direction algorithm^31^ (computation complexity is 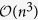) to solve the compressive sensing model^8^. Readers are referred to the Methods section for reconstruction details.

In the following experiments, 3000 spatially modulated random patterns (each owning 64 × 64 pixels) are sequentially projected onto the target scene. The frame rate of DMD is set to be 50 Hz, and the sampling rate of the bucket detector is 100 kHz. We utilize the novel self-synchronization technique in ref. 26 to synchronize the DMD and the detector. In all, it takes around 1 minute for data acquisition.

### Multispectral imaging results of MSPI

**Figure 3:**
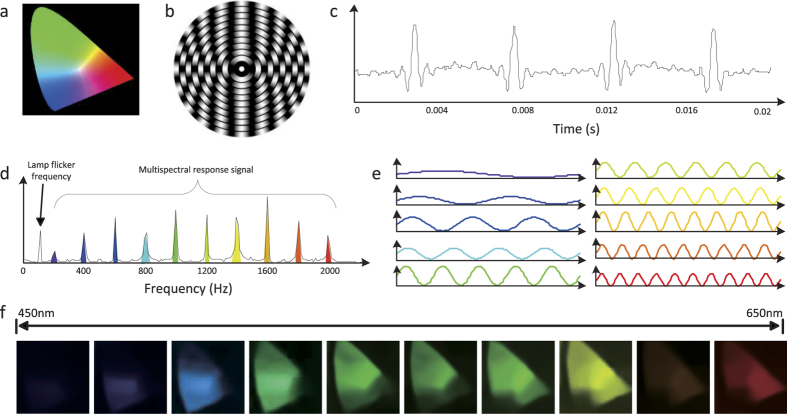
Multispectral imaging results of a color scene by MSPI. (**a**) is the target color scene, i.e., a printed film of the CIE 1931 color space. (**b**) is the spectral modulation film used in our setup. The rainbow spectrum is converged along the radius of the film, thus different wavelength bands are modulated with different sinusoidal periods as the film rotates. (**c**) shows an exemplar recorded 1D measurement sequence corresponding to a specific spatial pattern. (**d**) is the Fourier decomposition of the measurements, which exhibits several dominant frequencies. The coefficients of these dominant frequencies are exactly the response signals’ strengths of corresponding wavelength bands. (**e**) shows the decomposed sequences of different spectrum bands, and (**f**) presents the final reconstructed multispectral images (each owning 64 × 64 pixels) corresponding to 10 narrow bands ranging from 450 nm to 650 nm.

We first apply the proposed MSPI technique to capture multispectral images of a scene with rich color, to demonstrate its effectiveness. Here we use a printed “CIE 1931 color space” film (45 mm × 45 mm) owning a wide spectrum range (see Fig. 3(a)) as the target scene. In this experiment, the rainbow spectrum ranges from 450 nm to 650 nm, with the length being around 23 mm. We discretize the rainbow spectrum into 10 narrow bands by printing 10 2 mm annular rings onto the spectral modulation film, with their sinusoidal periods varying from 2 to 20 (as shown in Fig. 3(b)).

Given an exemplar spatial pattern, the recorded measurement sequence from the bucket detector is plotted in Fig. 3(c), and its corresponding Fourier coefficients are displayed in Fig. 3(d). One can see that there exist several dominant peaks in the Fourier domain, which locate at corresponding spectral modulation frequencies (the 60 Hz peak comes from lamp flicker due to voltage fluctuations). These peaks’ magnitudes are exactly the response signals’ strengths of corresponding spectrum bands. The other small fluctuations of the Fourier coefficients are caused by system noise. From this we can see that although these multispectral response signals are corrupted with system noise in the temporal domain, they are clearly distinguished in Fourier space. Therefore, we can easily demultiplex the multispectral response signals from each other and suppress system noise by a simple Fourier decomposition (see the Methods section for more details). The demultiplexed results are shown in Fig. 3(e). The frequencies match exactly with the sinusoidal annuluses printed on the film. After the spectral response signal demultiplexing, we can recover the target scene’s images at each wavelength band using a compressive sensing based algorithm. For spectral normalization, we place a Zolix OmniC 300 monochromator between the light source and the bucket detector as a filter to pre-calibrate the incident light’s spectrum and the detector’s spectral response (see Supplementary Figure 5), and use them to normalize the reconstructed images. The final reconstructed multispectral images are shown in Fig. 3(f), which have been integrated with the Canon EOS 5D MarkII camera’s RGB response curves^32^ (see Supplementary Figure 6) for better visualization. The positive results validate the effectiveness of the proposed MSPI.

### Quantitative analysis on the performance of MSPI

**Figure 4:**
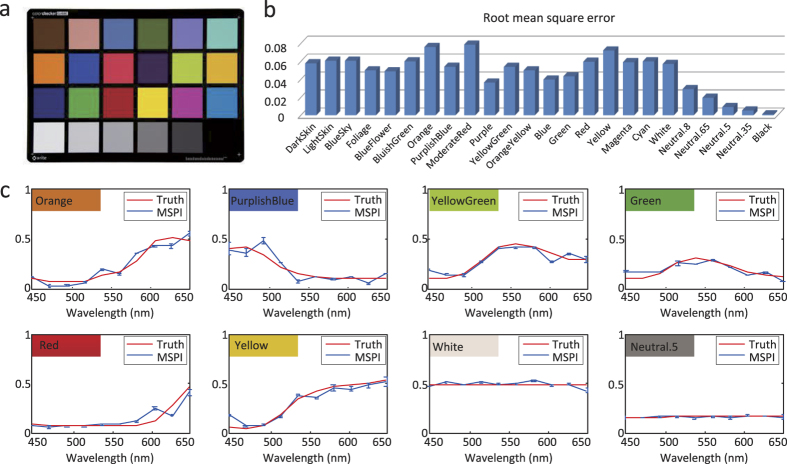
Quantitative analysis on the imaging accuracy of MSPI. (**a**) is the target scene—an X-Rite standard color checker, which consists of 24 swatches owning different spectra. We use MSPI to image the color checker and obtain 10 multispectral images (450 nm–650 nm). Then we calculate the average of all the pixels’ spectra in each swatch as the swatch’s final recovered spectrum. (**b**) presents the spectral reconstruction error of each swatch in terms of root mean square error. (**c**) shows direct comparison between the recovered spectra and their ground truth counterparts on several representative swatches. The standard deviation of each wavelength band is also calculated and denoted by blue bars. Both the small reconstruction error and deviation validate MSPI’s high imaging accuracy.

To quantitatively demonstrate the performance of MSPI, we acquire multispectral data of an X-Rite standard color checker (see Fig. 4(a)) using MSPI, and conduct quantitative analysis on the reconstruction accuracy. In the implementation, we introduce a pair of cylinder mirrors to match the shape of the incident light beam with that of the color checker (125 mm × 90 mm). For each swatch on the checker, we average all the pixels’ reconstructed spectra as the swatch’s reconstructed spectrum. The reconstruction error in terms of root mean square error among the 10 spectral bands is calculated for each swatch, and the results of all the 24 swatches are shown in Fig. 4(b). For more direct comparison, we show the spectrum comparison between the reconstruction and the ground truth of several representative swatches in Fig. 4(c). From the small deviations compared to the ground truth, especially the ones with relatively large reconstruction error (e.g., ‘Orange’ and ‘Yellow’), we can see that the reconstructed spectra of the swatches are well compliant with the ground truth. This experiment further validates the high reconstruction accuracy of MSPI, which benefits from the high precision of the spectral demultiplexing (clear-cut discrimination between the Fourier coefficients of signals and noise), as well as the optimization algorithm for reconstruction.

## Discussion

This paper proposes a novel multispectral imaging technique using a single bucket detector, termed as multispectral single pixel imaging (MSPI). Making use of the speed gap between slow spatial illumination patterning and fast detector response, MSPI extends conventional 2D spatial multiplexing to 3D spatial-spectral multiplexing via temporally sinusoidal spectral modulation within the elapse of each spatial pattern. This technique successfully resolves the target scene’s multispectral information without introducing additional acquisition time and computational complexity compared to conventional 2D SPI. It holds great potentials for developing cheap, compact and high-photon-efficiency multispectral cameras.

Specifications of the spectral modulation module in MSPI setups are flexible and can be easily customized. First, the width of the printed annuluses on the spectral modulation film can be adjusted for different spectral resolutions. Narrower annulus results in higher spectral resolution. Second, we can also use a diffraction grating with denser grooves to lengthen the rainbow stripe and thus raise the spectral resolution. One can refer to Supplementary Material 1.1 for more discussions about MSPI’s spectral resolution. Third, the spectral multiplexing mode can change easily by designing other film graphs. The sinusoidal spectral modulation utilized in current MSPI system is adopted due to its simplicity and robustness to noise. We refer readers to ref. 11 for more multiplexing methods.

Recalling that the proposed technique is a general scheme for multispectral imaging, it can be conveniently coupled with a variety of imaging modalities (both macroscopy and microscopy), by using corresponding suitable optical elements. Also, the scheme is wavelength independent, and thus can be applied to other spectrum ranges readily. This is especially important for the wavelengths under which array sensors are costly or unavailable. In addition, similar to the system in ref. 24, the spatial-spectral modulation can be conducted after the incident light interacts with the target scene. This enables us to analyze the scene’s spatial-spectral information without active illumination. One can refer to Supplementary Figure 7 for more details about MSPI under passive illumination, which is of much wider applicability.

Although MSPI owns many advantages over conventional multispectral imaging techniques, these benefits come at the expense of a number of projections as conventional SPI. In other words, MSPI makes a trade-off of temporal resolution for spatial and spectral resolution. As a reference, it takes us around 1 minute to project 3000 illumination patterns using our proof-of-concept MSPI setup, to produce a 10-channel 64*64-pixel multispectral data cube. To improve the spatial resolution, we can utilize the novel patterning strategies proposed in refs 14 and 33, where the authors show that projecting structural and adaptive patterns instead of random ones is beneficial for high spatial resolution imaging. Besides, the patterning strategies can also decrease projections and lower computation cost for enhanced temporal resolution. Further, the temporal resolution can be improved using a faster rotation motor or denser sinusoidal patterns for faster spectral modulation.

As for the algorithmic reconstruction, considering there exists abundant redundancy among different color channels^34^,^35^,^36^, we can utilize this cross channel prior in the algorithm to improve its reconstruction accuracy and reduce requisite projections. Also, since different wavelength bands are reconstructed separately in current MSPI, the reconstruction time can be shortened by utilizing graphics processing unit (GPU) to reconstruct different wavelength bands in a parallel manner.

## Methods

Reconstruction of the proposed MSPI technique consists of two main steps, namely spectral demultiplexing and multispectral reconstruction.

### Spectral demultiplexing

Due to the sinusoidal spectral modulation, for each spatially modulated pattern, its measurement sequence from the bucket detector consists of several response signals corresponding to different wavelength bands. These response signals own sinusoidal intensity variations of different periods. Thus in the Fourier domain, the measurement sequence presents coefficient peaks at corresponding dominant frequencies. Also, there exists system noise in the measurements, we assume which to be stochastic and zero-mean. In the Fourier domain, the noise mainly locates at high frequencies. Thus by applying simple Fourier decomposition^37^, we can separate the response signals from each other as well as the noise.

Mathematically, the Fourier decomposition describes a time series as a weighted summation of sinusoidal functions at different frequencies. A captured measurement sequence 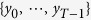 can be represented by a series of sinusoidal functions as


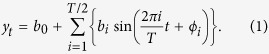


In this equation, 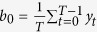, 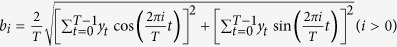, and 
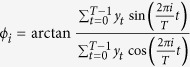
    . Specifically, *b*_0_ is the direct current component indicating the average of all the measurements, and *b*_*i*_ indicates the energy of the *i* th sinusoidal function at frequency 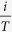. As stated before, each wavelength band corresponds to a specific sinusoidal modulation frequency. Thus, the above coefficients at the dominant frequencies are exactly the response signals corresponding to different wavelength bands. Here we adopt fast Fourier transform (FFT) to transfer the measurements into Fourier space, with computation complexity being 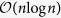. Then we demultiplex these response signals by finding local maximum coefficients around corresponding dominant frequencies.

By doing FFT to each measurement sequence of the spatial illumination patterns, we obtain a set of response signals for each wavelength band. Mathematically, assuming that the light at the wavelength *λ* is modulated with the sinusoidal frequency 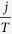, we can obtain a response signal *b*_*j*_ from the measurement sequence corresponding to one projecting pattern. Considering that we project *m* patterns, we get *m* response signals of the wavelength *λ*. In the following, we indicate the response signal set as a row vector 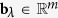. Each entry in **b**_*λ*_ corresponds to a response signal of the band *λ* for one pattern.

### Multispectral reconstruction

After demultiplexing the response signals of different wavelengths, the subsequent multispectral reconstruction is implemented separately in each wavelength band. In the reconstruction, we assume the spatial pixel number of each illumination pattern is *n*, and denote the pattern set as 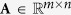 (each pattern is represented as a row vector in the matrix). The reconstructed multispectral images own the same spatial resolution as the illumination patterns, and are denoted as 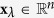 for the wavelength *λ*. With the above denotations, the multispectral reconstruction is performed by solving the following optimization problem^8^:


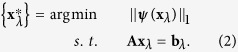


The definition of the objective function comes from a sparsity prior: natural images are statistically sparse when represented with an appropriate basis set (e.g. the discrete cosine transform basis)^38^. We use ***ψ***(**x**_*λ*_) to denote the coefficient vector, and aim to minimize its *l*_1_ norm to force its sparsity. Note that *ψ* is the mapping operator to the transformed domain. The constraint is the formation of the response signals. Equation (2) is a standard *l*_1_ optimization problem, and we solve it using the linearized alternating direction method^31^ considering its satisfying performance (computation complexity is 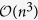). This results in the reconstructed scene image corresponding to the specific wavelength band *λ*. After applying the above reconstruction to all the wavelength bands, we get multispectral images of the target scene. Note that these reconstructed multispectral images consist of three spectral components including the illumination’s spectrum, the scene’s spectrum and the bucket detector’s spectral response. Therefore, to produce final multispectral images of the scene, the images need to be normalized by both the pre-calibrated spectrum of the illumination and the spectral response of the detector. Here we place a Zolix Omni–*λ* 300 monochromator as a light filter between the light source and the bucket detector for the pre-calibration. We refer readers to Supplementary Material 3 and Supplementary Figure 5 for more details.

## Additional Information

**How to cite this article**: Bian, L. *et al*. Multispectral imaging using a single bucket detector. *Sci. Rep.* **6**, 24752; doi: 10.1038/srep24752 (2016).

## Supplementary Material

Supplementary Information

Supplementary video 1

Supplementary video 2

## Figures and Tables

**Figure 1:**
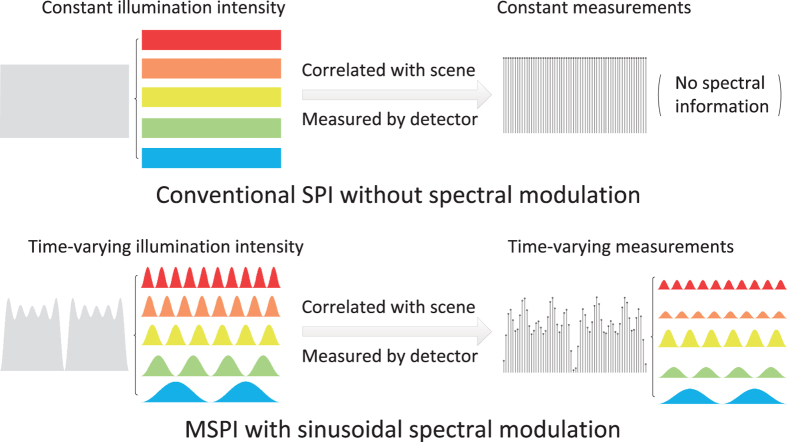
Illumination’s difference between conventional single pixel imaging (SPI) and the proposed multispectral single pixel imaging (MSPI). Due to the response speed gap between a bucket detector (MHz or GHz) and a spatial light modulator (no higher than KHz), the detector can collect a dense sequence of measurements during the elapse of each spatially modulated illumination pattern. In conventional SPI, given a spatial pattern, its light intensity and corresponding measurements are constant. Thus no spectral information can be extracted from the measurement sequence. Differently, both the illumination’s intensity and the measurements are time-varying in MSPI, because the light’s intensity of each spectral component changes sinusoidally over time with different periods. Thus we multiplex the target scene’s spectral information into the measurement sequence during the elapse of each spatial pattern, and can use Fourier decomposition to demultiplex these spectral information. This is the main difference between conventional SPI and the proposed MSPI.
