# Supplementary material for: Multispectral imaging using a single bucket detector
Source: Sci Rep. 2016 Apr 22;6:24752. doi: 10.1038/srep24752 (PMC4840436; doi:10.1038/srep24752)
Supplement: Supplementary Information [file srep24752-s1.pdf]

Supplementary materials of
Multispectral imaging using a single bucket detector

Liheng Bian[1], Jinli Suo[1,*], Guohai Situ[2], Ziwei Li[1], Jingtao Fan[1], Feng Chen[1] and Qionghai Dai[1]

[1]*Department of Automation, Tsinghua University, Beijing 100084, China*

[2]*Shanghai Institute of Optics and Fine Mechanics, Chinese Academy of Sciences, Shanghai 201800, China*

[*]*jlsuo@tsinghua.edu.cn*

1 System Specifications and Performance Analysis

1.1 Spectral resolution of MSPI

The spectral resolution of MSPI is determined by the length of the rainbow spectrum and the width of each annulus on the printed film. To be more specific, a longer rainbow spectrum and narrower printed annulus would result in higher spectral resolution.

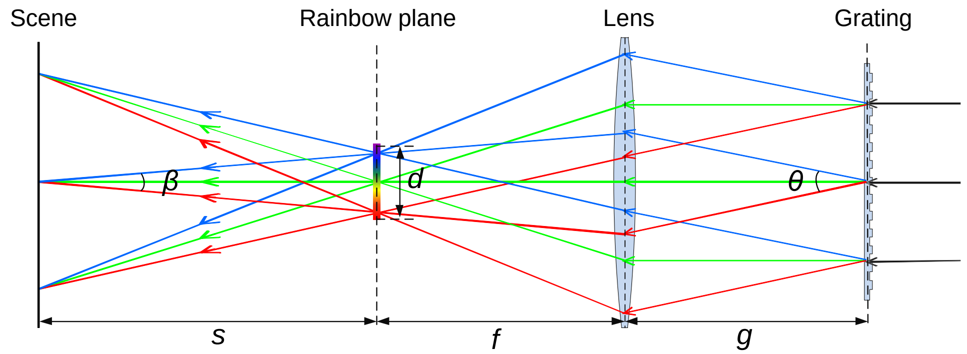

Supplementary Figure 1: **Light path of the spectral modulation module in MSPI.**

For the length of rainbow spectrum, it is related to specific optical elements used in the setup. In the following, we analyze the related factors mathematically. To simplify the analysis and make it independent of the spatial modulation module, we assume the incident light onto the diffraction grating is parallel light and normal to the grating, as shown in Supplementary Figure 1. Thus, according to optical geometry, the distance between the lens and the rainbow plane is the lens's

focal length, namely f, and we can also get

$$\frac{1}{s+f}+\frac{1}{g}=\frac{1}{f}, \tag{1}$$

where s is the distance between the target scene and the rainbow spectrum, and g is the distance between the lens and the diffraction grating.

Then, utilizing paraxial approximation, we get

$$(s+f)\beta = g\theta \tag{2}$$

and

$$\beta = \frac{d}{s}, \tag{3}$$

where β is the converging angle of the illumination onto the scene, θ is the diffraction angle of the grating, and d is the length of the rainbow spectrum.

Integrating both Supplementary equation (1) and Supplementary equation (2) into Supplementary equation (3), we obtain

$$d = f\theta. \tag{4}$$

From Supplementary equation (4) we can see that the length of the rainbow spectrum is determined by the lens's focal length and the grating's diffraction angle. The diffraction angle is further determined by its groove number. To conclude, longer focal length and denser grating would produce longer rainbow spectrum. As a reference, we used a 200mm focal length lens and a 600 grooves/mm grating in our setup, which produced a 23mm rainbow spectrum covering the visible wavelength from 450nm to 650nm.

However, one should note that the rainbow spectrum owns a physically limited spectral resolution caused by grating's diffraction mechanism, meaning that the resolved rainbow spectrum is not continuous [1,2]. The resolving power of a diffraction grating is given by [2]

$$R = \frac{\lambda}{\Delta\lambda} = mN, \tag{5}$$

where λ is the light's wavelength, $\Delta\lambda$ is the minimum wavelength interval that the grating can resolve around λ, m is the diffraction order, and N is the total number of grooves illuminated. In

our setup, $m = 1$, $N \approx 600 \times 25 = 15000$ grooves. Thus, the resolving power of the rainbow spectrum is around 15000. As a reference, at the wavelength of $\lambda = 650nm$, the physical spectral resolution of the rainbow spectrum is $\Delta\lambda = \frac{\lambda}{R} = \frac{650}{15000} \approx 0.04nm$.

For the width of the printed annulus, it is determined by printer's printing precision, which varies from hundreds dpi (dots per inch) to thousands dpi. In our system, the printing resolution is 1200×1200 dpi. Recalling that the rainbow spectrum is 23mm long and ranges from 450nm to 650nm, thus our system can produce a theoretical spectral resolution of $\frac{200}{23}/\frac{1200}{25.4} \approx 0.18$nm.

1.2 Spatial resolution (in pixels) of reconstructed images

Physically, the upper limit of MSPI's spatial resolution for the reconstructed multispectral images is determined by the adopted spatial light modulator (SLM), because the reconstructed images own the same pixel number as that of the spatially modulated patterns. In our setup, we use a digital micromirror device (DMD, Texas Instrument DLP Discovery 4100 DLP7000) owning 1024×768 micro-mirrors for spatial modulation, thus the maximum spatial resolution of our MSPI setup is also 1024×768 pixels. One can choose other SLM devices for different spatial resolutions. For example, the spatial resolution of Texas Instrument DLP LightCrafter DLP9000 is 2560×1600. We refer readers to Ref. [3] for more DMD specifications.

In practice, the pixel number is often much lower than the upper limit to tradeoff for higher frame rate. This is caused by the imaging mechanism of the utilized single pixel imaging technique: retrieving scene's spatial information via its correlated measurements with a number of illumination patterns. For example, in our experiment, it needs 3000 spatially random modulated projections to reconstruct 64×64 pixel images, and the acquisition takes round 1 minute. If one wants to achieve higher frame rate with a similar reconstruction quality, the spatial resolution must be lowered to reduce requisite projections. We refer readers to Ref. [4] for more discussions about the tradeoff between pixel number and frame rate in single pixel imaging.

Besides, as stated in the discussion section, projecting structural patterns instead of random ones can largely improve the spatial resolution while decreasing projections and lowering computation cost [5,6]. Here we demonstrate this using the technique in Ref. [5]. We sequentially project four phase-shifted sinusoidally modulated (in spatial space) patterns (each owning 256×256 pixels) to sample each spatial frequency of the scene's spatial spectrum, and measure 30% of the entire spa-

tial spectrum which is sufficient for visually promising reconstruction as stated in Ref. [6]. The total projected pattern number is around 39000, and the reconstructed multispectral images are shown in Supplementary Figure 2. From the results we can see that the flower details are reconstructed well, which validates the advantages of structural patterns for high resolution imaging, and the flexibility for spatial resolution improvement of our approach.

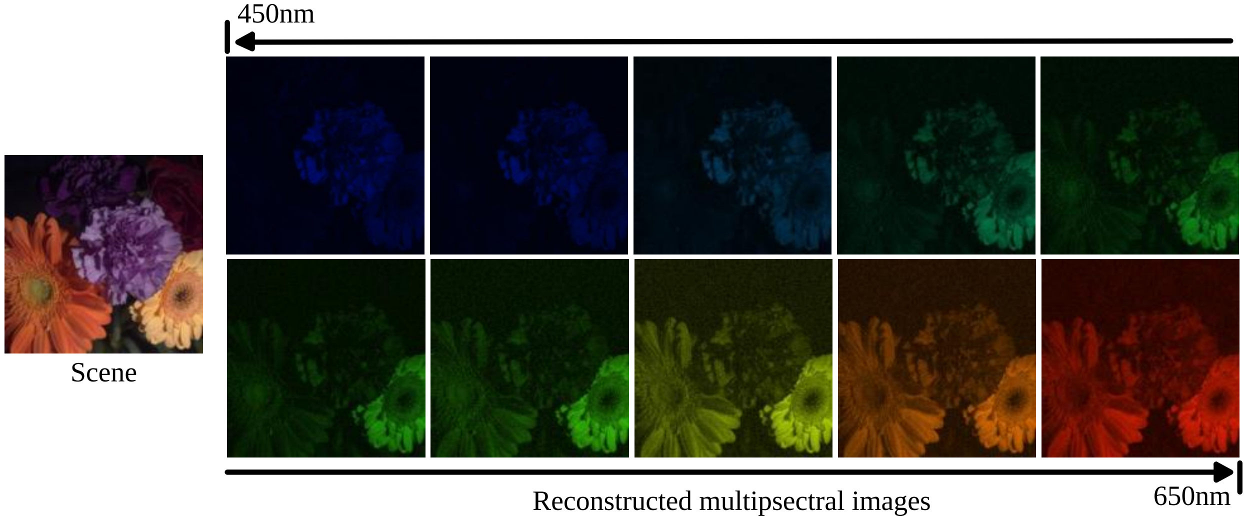

Supplementary Figure 2: **MSPI results using sinusoidally spatial modulation.**

1.3 Photon efficiency of MSPI

There are three components in the MSPI setup that would affect the system's photon efficiency in terms of the ending detection unit, including the spatial light modulator, the optical diffraction grating, and the spectral modulation film.

At the spatial light modulator, since we utilize random binary patterns, there are around half of the photons emitted from the light source are filtered, resulting in decreased photon efficiency by around 1/2. The reflection efficiency of the utilized DMD in the visible wavelength range is higher than 91%, so the spatial modulation would block round 55% photons in total.

As for the diffraction grating, we use a 600 grooves/mm transmission grating in our proof-of-concept setup, whose light efficiency is around 50% [1] (see Supplementary Figure 3 for specific efficiency curves). To maintain high light efficiency, one can use reflective blazed gratings instead, which can concentrate more of light's energy (up to 80%) on the first order spectrum and thus own higher light efficiency. We refer readers to Supplementary Figure 4 for more details about the

diffraction efficiency of reflective blazed gratings [7,8].

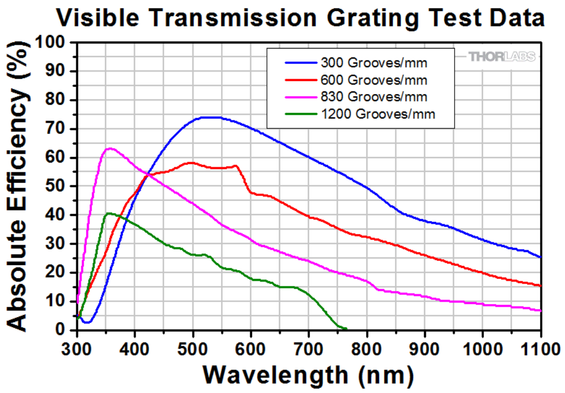

Supplementary Figure 3: **Diffraction efficiency of the utilized transmission grating.**

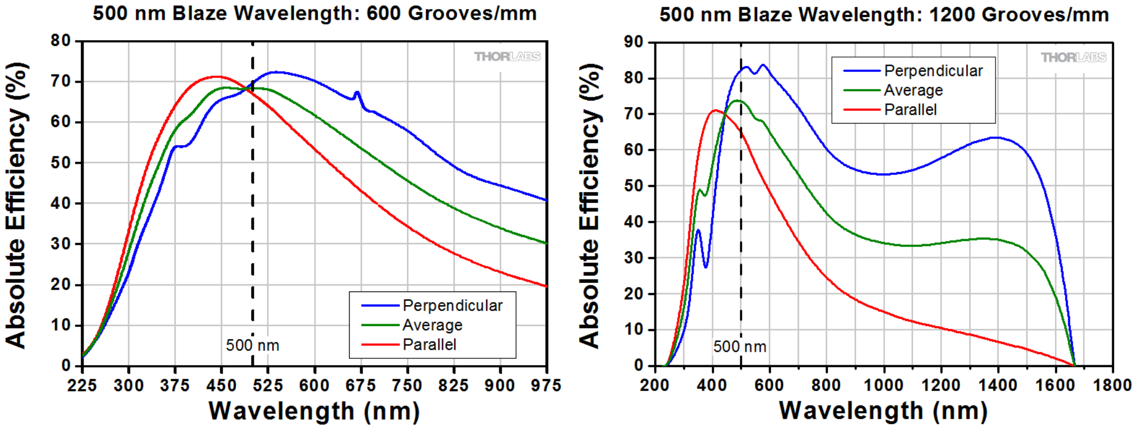

(a) (b)

Supplementary Figure 4: **Diffraction efficiency curves of two reflective blazed gratings with the blaze wavelength being 500nm.** (a) corresponds to a grating of 600 grooves/mm, while (b) shows the efficiency of a grating of 1200 grooves/mm.

After passing the spectral modulation film, around half of the photons are filtered out due to the utilized sinusoidal modulation, i.e., the light efficiency is again halved.

Taking the above three factors comprehensively, the photon efficiency of our proof-of-concept MSPI system from the incident illumination to the ending detection unit is around 1/8. The efficiency can be raised up to around 1/5 using a reflective blazed grating with denser grooves, and can be further increased by utilizing high photon-efficiency modulation codes.

Compared to conventional multispectral imaging techniques, the increased photon efficiency of MSPI comes from two aspects. One is that we use the single pixel imaging scheme for spatial multiplexing, and another is that we sinusoidally modulate the illumination's spectrum for spectral multiplexing. We refer readers to Ref. [9] for detailed and quantitative analysis of illumination multiplexing's advantages for improved light efficiency.

References

1. Thorlabs. Visible transmission gratings. `http://www.thorlabs.us/newgrouppage9.cfm?objectgroup_id=1123`. [Online; accessed 29-Oct-2015].

2. Palmer, C. A., Loewen, E. G. & Thermo, R. *Diffraction grating handbook* (Newport Corporation Springfield, Ohio, USA, 2005).

3. Edgar, M. P. *et al.* Simultaneous real-time visible and infrared video with single-pixel detectors. *Sci. Rep.* **5** (2015).

4. Instruments, T. TI DLP technology overview. `http://www.ti.com/lsds/ti/analog/dlp/overview.page`. [Online; accessed 29-Oct-2015].

5. Zhang, Z., Ma, X. & Zhong, J. Single-pixel imaging by means of fourier spectrum acquisition. *Nat. Commun.* **6** (2015).

6. Bian, L., Suo, J., Hu, X., Chen, F. & Dai, Q. Fourier computational ghost imaging using spectral sparsity and conjugation priors. *arXiv preprint arXiv:1504.03823* (2015).

7. Thorlabs. Visible ruled reflective diffraction gratings. `http://www.thorlabs.us/newgrouppage9.cfm?objectgroup_id=8626`. [Online; accessed 29-Oct-2015].

8. Thorlabs. Diffraction gratings. `http://www.thorlabs.us/navigation.cfm?guide_id=9`. [Online; accessed 29-Oct-2015].

9. Schechner, Y. Y., Nayar, S. K. & Belhumeur, P. N. A theory of multiplexed illumination. In *IEEE Int. Conf. Comput. Vision*, 808–815 (2003).

2 Supplementary Figures

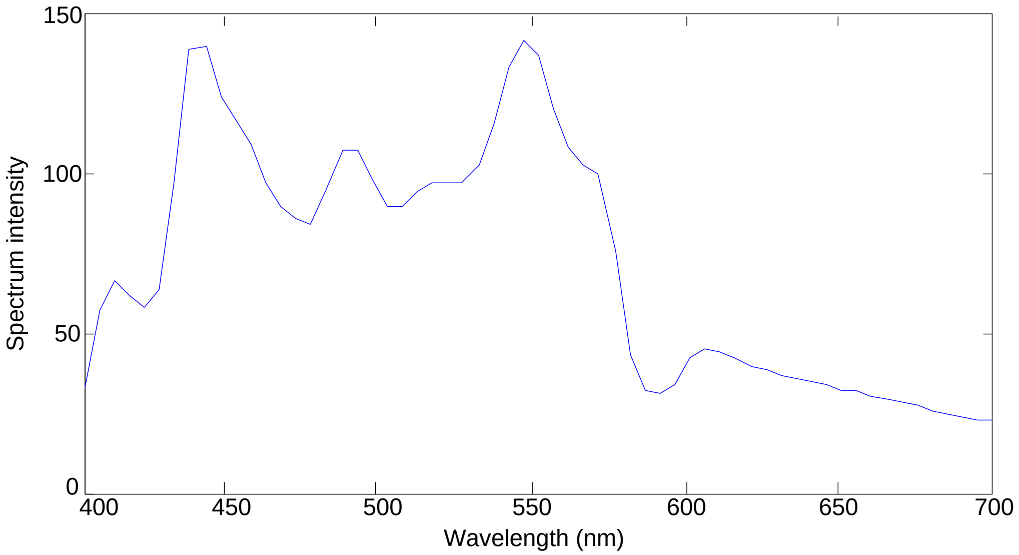

Supplementary Figure 5: **Pre-calibrated spectrum of the utilized light source using a Zolix Omni–λ 300 monochromator.** We use the monochromator as a light filter between the light source and the bucket detector, to measure the light's spectrum intensity at each wavelength. We vary the wavelength from 400nm to 700nm, with a step being 5nm. The measured spectrum is the product of the illumination's spectrum and the detector's spectral response, and is utilized to normalize the reconstructed multispectral data at each spatial point.

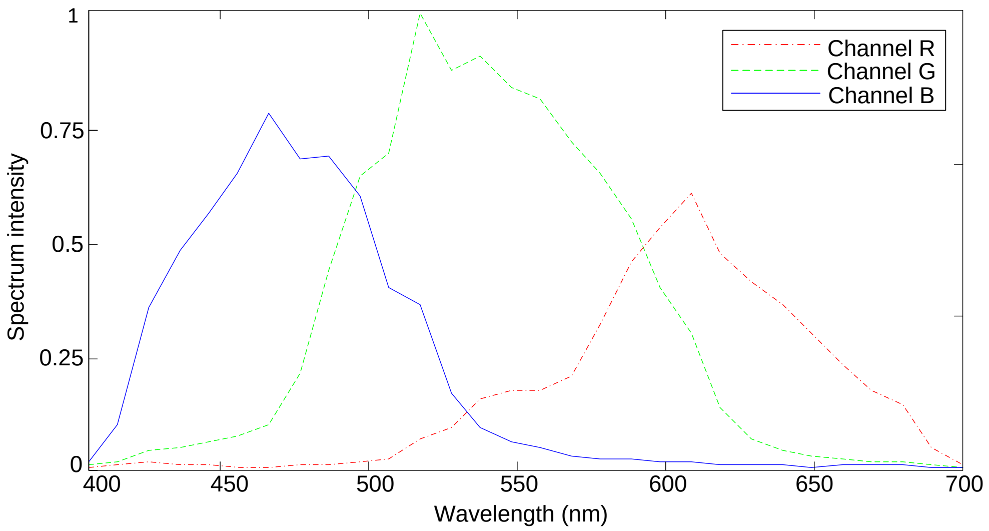

Supplementary Figure 6: **RGB response curves of the Canon EOS 5D MarkII camera.** We distribute the pixel intensities of reconstructed multispectral images at each spatial point into RGB channels using the RGB ratio of the response curves at corresponding wavelength. This provides color visualization of the 10 reconstructed multispectral images, as shown in Figure 3(f) in the main paper.

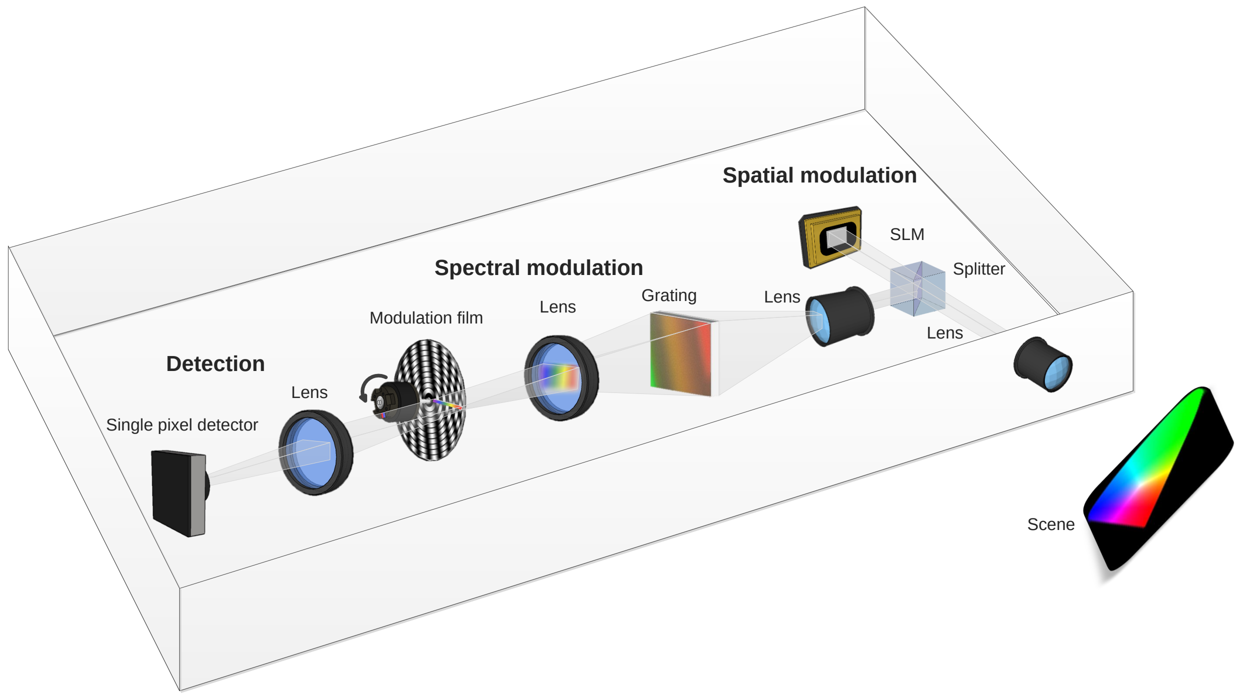

Supplementary Figure 7: **An alternative MSPI system without active light source.** We place both the spatial and spectral modulation modules after the light interactes with the target scene. This enables us to integrate the modulation module and the detection module together as a whole, to analyze both the scene's spatial and spectral information as an integrated multispectral imager without active light source.

3 Reconstruction Derivation

Reconstruction of the proposed MSPI technique consists of two main steps, namely spectral demultiplexing and multispectral reconstruction.

3.1 Spectral demultiplexing

For spectral demultiplexing, we first apply discrete Fourier transform to the measurement sequence of each pattern, to transfer them into Fourier space. Mathematically, we denote the measurement sequence of a pattern as $\mathbf{s} \in \mathbb{R}^r$, where r is the number of measurements. Then, discrete Fourier transform is performed as

$$\widetilde{\mathbf{s}} = \begin{bmatrix} \omega^{0\times 0}, & \omega^{0\times 1}, & \cdots & \omega^{0\times(r-1)} \\ \omega^{1\times 0}, & \omega^{1\times 1}, & \cdots & \omega^{1\times(r-1)} \\ \vdots & \vdots & \ddots & \vdots \\ \omega^{(r-1)\times 0}, & \omega^{(r-1)\times 1}, & \cdots & \omega^{(r-1)\times(r-1)} \end{bmatrix} \mathbf{s} \tag{6}$$

where $\omega = e^{\frac{-2\pi i}{r}}$. Here we utilize the default fft function in Matlab to conduct the transform.

With the Fourier coefficients determined, the response signals corresponding to different wavelength bands can be demultiplexed by finding the local maximum coefficients around each corresponding frequency. Specifically, utilizing the electric motor's rotation speed and the film's modulation periods, we can easily determine the specific spectral modulation frequencies corresponding to each wavelength band. Considering actual deviation of the motor's rotating speed, we extract the response signals at each wavelength band by searching for the local maximum coefficients around their corresponding frequencies in the Fourier domain.

3.2 Multispectral reconstruction

As the response signals of each wavelength band determined, we move on to multispectral reconstruction. In the following, we present the specific model derivation.

3.2.1 Variable definition

All the mathematical variables and their physical meanings are summarized below.

m	Number of the spatial patterns;
n	Pixel number of each pattern;
k	Number of the bases for sparse representation;
$\mathbf{A} \in \mathbb{R}^{m \times n}$	Pattern set;
$\mathbf{x} \in \mathbb{R}^{n \times 1}$	Scene image at a specific wavelength;
$\mathbf{b} \in \mathbb{R}^{m \times 1}$	Response signals corresponding to the specific wavelength;
$\mathbf{B} \in \mathbb{R}^{n \times k}$	Representation basis set;
$\mathbf{c} \in \mathbb{R}^{k \times 1}$	Representation coefficient vector.

3.2.2 Model derivation

As presented in the main paper, the reconstruction model is

$$\begin{aligned} \{\mathbf{x}^*\} = \arg\min \quad & ||\psi(\mathbf{x})||_1 \\ s.t. \quad & \mathbf{Ax} = \mathbf{b}. \end{aligned} \tag{7}$$

In the above model, ψ is the mapping operator used to transform an image into a specific representation domain, and $\psi(\mathbf{x}_\lambda)$ denotes the coefficient vector. $||\cdot||_1$ is the l_1 norm that for any vector $\mathbf{u} = [u_1, u_2, \cdots, u_m]$, i.e., $||\mathbf{u}||_1 = \sum_{i=1}^{m} |u_i|$. To make it easier to understand, we use $\mathbf{B}$ to denote the representation basis, and $\mathbf{c}$ as the corresponding coefficient vector (namely $\mathbf{c} = \psi(\mathbf{x})$). Thus, we get $\mathbf{x} = \mathbf{Bc}$, and transform the above model to

$$\begin{aligned} \{\mathbf{c}^*\} = \arg\min \quad & ||\mathbf{c}||_1 \\ s.t. \quad & \mathbf{ABc} = \mathbf{b}. \end{aligned} \tag{8}$$

In the above model, $\mathbf{A}$, $\mathbf{B}$ and $\mathbf{b}$ are all known. Here we use $\mathbf{D} = \mathbf{AB}$ to incorporate these two linear operations, and the model becomes

$$\begin{aligned} \{\mathbf{c}^*\} = \arg\min \quad & ||\mathbf{c}||_1 \\ s.t. \quad & \mathbf{Dc} = \mathbf{b}. \end{aligned} \tag{9}$$

This is a standard l_1 optimization problem, and we solve it using the linearized alternating direction method considering its satisfying performance. After obtaining the optimal $\mathbf{c}^*$, we multiply it with the representation basis $\mathbf{B}$ to obtain the reconstructed scene image corresponding to the specific

wavelength band. By conducting the above reconstruction in each wavelength band separately, we get reconstructed multispectral images of the target scene.

One should note that the above reconstructed multispectral images consist of three spectral components including the illumination's spectrum, the scene's spectrum and the bucket detector's spectral response. Therefore, to produce final multispectral images of the scene, we need to normalize the above reconstructed images with the pre-calibrated spectrum of the illumination and the spectral response of the detector. Here we use a Zolix Omni–λ 300 monochromator as a light filter between the light source and the bucket detector to measure the light's spectrum intensity at each wavelength for the pre-calibration. We vary the wavelength of the monochromator from 400nm to 700nm, with a step being 5nm. The measured spectrum is exactly the product of the illumination's spectrum and the detector's spectral response (see Supplementary Figure 5 for more information).

4 Supplementary video legends

Supplementary video 1.

This supplementary video introduces the proposed multispectral single pixel imaging (MSPI) system, and demonstrates what optical elements the system consists and how they work.

Supplementary video 2.

This supplementary video shows the illumination's spatial-spectral multiplexing and demultiplexing in MSPI.
